# In Vitro Maturation of Cumulus–Oocyte Complexes and In Vitro Sperm Capacitation Significantly Increase the Expression and Enhance the Location of the CXCL12 and CXCR4 Anchoring Attractant Complex in Pigs

**DOI:** 10.3390/ani11010153

**Published:** 2021-01-11

**Authors:** Cristina A. Martinez, Manuel Alvarez-Rodriguez, Maite Casado-Bedmar, Heriberto Rodriguez-Martinez

**Affiliations:** 1 Department of Biomedical & Clinical Sciences (BKV), BKH/Obstetrics & Gynaecology, Faculty of Medicine and Health Sciences, Linköping University, SE-58185 Linköping, Sweden; manuel.alvarez-rodriguez@liu.se (M.A.-R.); heriberto.rodriguez-martinez@liu.se (H.R.-M.); 2 Department of Biomedical & Clinical Sciences (BKV), KOO/Surgery, Orthopedics and Oncology, Faculty of Medicine and Health Sciences, Linköping University, SE-58185 Linköping, Sweden; maite.casado@liu.se

**Keywords:** sperm chemoattractants, cytokine-receptor complexes, capacitation, COCs, fertilization, pig

## Abstract

**Simple Summary:**

The process of mammalian fertilization is dependent on many mechanisms mediated by regulatory genes and proteins expressed in the gametes and/or the female genital tract. This study aimed to determine the expression and location of the cytokine complex CXCL12:CXCR4 in the porcine gametes: oocytes and spermatozoa. This complex is known to play a pivotal role for sperm attraction towards the oocyte prior to internal fertilization in several mammalian species. Gene and protein expressions were analyzed in female and male porcine gametes. The results showed that the CXCL12 gene expression was higher in mature cumulus cells, and CXCR4 was higher in capacitated spermatozoa, both being requisites for gametes to accomplish fertilization. Moreover, for the first time, the CXCL12 protein was located in the cytoplasm of cumulus cells from mature COCs, and the CXCR4 protein was expressed in the midpiece and principal piece of uncapacitated spermatozoa and also in the sperm head of capacitated spermatozoa. These findings increase our current knowledge on porcine physiology of fertilization and reproduction, leading to possible improvements in the performance of reproductive technologies.

**Abstract:**

Successful internal fertilization in mammals depends on several mechanisms, including those triggering the so-called “sperm attraction” towards the oocyte, which include some oocyte-derived sperm chemoattractants and interactive protein complexes, such as the cytokine C-X-C motif chemokine 12/C-X-C chemokine receptor type 4 (CXCL12-CXCR4) receptor complex. The presence and precise localization of these crucial proteins was determined hereby, for the first time, in porcine cumulus–oocyte complexes (COCs) and spermatozoa. CXCL12 was overexpressed in the cumulus cells of in vitro matured, compared to immature COCs (*p* < 0.05), with its receptor (CXCR4) being up-regulated in capacitated spermatozoa (*p* < 0.03) compared to uncapacitated spermatozoa. The CXCR4 appeared specifically localized in the sperm tail of non-capacitated spermatozoa and also in the sperm head of capacitated spermatozoa, suggesting that the CXCL12-CXCR4 signaling complex would play a pivotal role in attracting capacitated spermatozoa towards the oocyte, facilitating fertilization in pigs.

## 1. Introduction

In vivo fertilization involves a series of different events that, in mammals, includes sperm capacitation, oocyte nuclear and cytoplasmic maturation, gamete transport to the site of fertilization in the oviduct, and sperm binding to the zona pellucida (ZP), among others [1,2]. Chemotaxis is considered one of the most important factors guiding spermatozoa towards the oocyte–cumulus complex (COC) and involves hormones such as progesterone, glycosaminoglycans (GAGs), odorants, etc., although it remains unclear how the final interaction steps occur [3,4,5]. Some of these “distant” chemoattractants are known to activate downstream pathways, increasing intracellular cAMP concentrations and the influx of Ca^2+^ in spermatozoa, issuing hyperactivation and somehow guiding the presumably capacitated cells towards the oocyte [6,7]. Other chemotactic factors are present in the oocyte ZP and reportedly, in some species, attract advanced spermatozoa, despite the presence of a major coating cloud of expanded cumulus cells in species such as the pig. In pigs, as in other mammalian species, the ZP is, prior to fertilization, still embedded in the hyaluronic acid-expanded matrix which spermatozoa must traverse, dislodging the cumulus cells to reach the ZP [8,9]. The cumulus cells might therefore play dual roles by forming a massive cloud that spermatozoa can easily find or by mechanically entrapping them but also releasing factors relevant to sperm–oocyte interaction [10,11].

Upon arrival, some ZP-glycoproteins (like ZPB/ZP4 and ZPC/ZP3) control sperm recognition and primary binding via N- and O-glycans, which attach to the ZP-glycoproteins in many mammalian species [10,12]. Moreover, previous studies have emphasized the role of integrins (αvβ1 and β1) in the recognition of the sperm protein ADAM-2 in mammals [11,13].

The C-X-C motif chemokine 12 (CXCL12, also called stromal derived factor (SDF-1)) was suggested as an important chemoattractant of bone marrow-derived mesenchymal stem cells modulating immunoregulatory actions and promoting angiogenesis [14,15]. Moreover, CXCL12 modulates T cell migration within the pregnant uterus by binding its ligand C-X-C chemokine receptor type 4 (CXCR4) through chemoattraction and by establishing a beneficial microenvironment for the fetus [16,17]. This CXCL12-CXCR4 interaction is also known to promote cell–cell interaction in the embryo–endometrium interface by activating several endometrial proteins belonging to the integrin family, mostly alpha3, alpha5, beta1, and beta3 subunits, accompanied by an increased phosphorylation of ERK1/2, JNK, and the p38 pathway [18,19]. Dysregulation of CXCL12-CXCR4 signaling has been associated with placental dysfunction by jeopardizing trophoblast invasion and migration, leading to pregnancy disorders like preeclampsia, miscarriage, and fetal growth restriction [20].

CXCL12 was previously reported to be produced by cumulus cells from human [21], cattle [22], and sheep oocytes [23], with its receptor (CXCR4) being localized in the head or tail of human [24] and bovine [22] spermatozoa.

The present study, considering the wide presence of cytokines in the female genital tract [25], postulates that such a CXCL12/CXCR4 complex is also present in the COC-sperm scenario in the porcine species, where it could serve as an anchoring attractant for spermatozoa. Whether such a complex is present, whether the expression of its components depends on oocyte maturation (CXCL12) or sperm capacitation (CXCR4), respectively, and where they are precisely located constitute the objectives of this study.

## 2. Materials and Methods

### 2.1. Animals

All experiments involving animals followed the regulation of the European Directive 2010/63/EU and were examined and approved prior to experiments by the Ethical Committee for Experimentation with Animals of Murcia University (research code: 522/2019), and in compliance with current Swedish legislation (SJVFS 2015:24). The experiments were approved in advance by the “Regional Committee for Ethical Approval of Animal Experiments” (Linköpings Djurförsöksetiska nämnd) in Linköping, Sweden (permits no. 75–12, no. ID1400 and Dnr 03416-2020 (26 March 2020).

### 2.2. Experimental Design

In order to study the gene and protein expression of CXCL12, pools of 10 immature COCs (*n* = 3), pools of 10 in vitro matured COCs (*n* = 3), and pools of >100 cumulus cells from in vitro matured COCs (*n* = 3) were collected for qPCR and immunofluorescence analyses. Furthermore, spermatozoa were collected from semen pools (*n* = 3), where the ejaculates from three different mature, fertile breeding boars (Köttböndernas, Hållsta, Sweden) constituted each of the pools. Spermatozoa were in vitro capacitated and subjected to qPCR and immunofluorescence analyses.

### 2.3. Oocyte Collection and In Vitro Maturation

Medium-sized follicles (3–7 mm in diameter) collected from pre-pubertal gilts at a local slaughterhouse were cut with a sterile scalpel blade in TL-HEPES-PVA for COCs recovery. Only the COCs containing two or more layers of compact cumulus cells were selected for this experiment. Groups of 70–75 COCs were matured in a four-well multi-dish (Nunc, Roskilde, Denmark) containing 500 μL of maturation medium (TCM-199, Sigma-aldrich, Madrid, Spain) supplemented with 10 IU/mL eCG (Folligon, Intervet International B.V. Boxmeer, The Netherlands) and 10 IU/mL hCG (Veterin corion, Divasa Farmavic S.A. Barcelona, Spain) for 22 h. The COCs were then incubated for an additional 22 h in the same medium without hormones. All steps were performed under paraffin oil at 38 °C in 5% CO_2_ in air and 95–97% relative humidity. At 44 h of maturation, the COCs were washed in phosphate-buffered saline (PBS) and stored in 5 μL PBS at −80 °C until further analyses.

### 2.4. Cumulus Cells Handling

After oocyte maturation, the cumulus cells suspended in a maturation medium with 0.1% hyaluronidase were removed by vortexing for 2 min at 1660 rounds/min. The cumulus cells were then washed in PBS, centrifuged at 12,000× *g* for 10 min, and stored in 5 μL PBS at −80 °C until further analysis.

### 2.5. Sperm Handling and In Vitro Capacitation

Boar ejaculates were supplied as commercial artificial insemination (AI) doses (6 batches) from Svenska Köttföretagen AB, Hållsta, Sweden, extended in Hampshire Longlife to 4.8 × 10^9^ total spermatozoa/dose. Each batch contained a pool of three different breeding boars of proven fertility and semen quality (>80% motility, <15% total sperm abnormalities) and was stored at 17 °C temperature, as recommended for AI purposes. All incoming sperm samples were re-examined for motility (QualiSperm, Biophos AG, Pfäffikon, Switzerland) [26] and sperm viability using Propidium iodide labelling [27] right before use. Values >80% were consistently found for proportions of motile and viable spermatozoa. Spermatozoa were washed twice in phosphate-buffered saline (PBS, 200× *g*, 10 min) and incubated in a capacitation medium (37 mm NaHCO_3_, 2.25 mm CaCl_2_, 2 mm caffeine, 0.5% bovine serum albumin, and 310 mM lactose) at 38 °C, 5% CO_2_ for 30 min [28]. Following centrifugation (10,000× *g*, 10 min), the sperm pellets were stored at −80 °C until further analysis.

### 2.6. RNA Isolation and Reverse Transcription

Total RNA from the COCs, cumulus cells, and spermatozoa (100 × 10^6^ spermatozoa in total) was extracted using a RNeasy micro kit (Qiagen, Venlo, The Netherlands) and the quantity was measured using a NanoDrop system (Thermo Scientific, Fremont, CA, USA). Then, the RNA was reverse-transcribed to cDNA using the High-Capacity cDNA Reverse Transcription Kit (Applied Biosystem, Foster City, CA, USA) at 25 °C for 10 min and 37 °C for 120 min, followed by 85 °C for 5 s in a Thermal cycler (BioRad-DNA Engine, Hercules, CA, USA).

### 2.7. Real-Time Quantitative Polymerase Chain Reaction (RT-qPCR)

The relative mRNA expression of CXCL12 and CXCR4 in the COCs and spermatozoa, respectively, was quantified by q-PCR using the Real-Time PCR Detection System (CFX9; Bio-Rad Laboratories, Inc.; Berkeley, CA, USA). Primers for the *CXCL12*, *CXCR4*, and *GAPDH* (housekeeping) genes were designed with the software primer3 and Blast [29]. The primers were designed to cross exon–exon boundaries to ensure that the amplified product was not generated from genomic DNA contamination. For increased sensitivity, *GAPDH* primers were specially designed to avoid differentiation between the isoforms. The specificity of the primers was checked using a BLAST analysis against the genomic NCBI database. The primer pairs details are listed in Table 1. PowerUp SYBR Green Master Mix (2X) (Applied Biosystems, Foster City, CA, USA) was used for PCR reactions. The final reaction volume was 10 μL (2 μL of cDNA (25 ng), 5 μL of 2X Master mix, 1 μL of each primer (500 nM), and 1 μL of dH_2_O). The following PCR conditions were used: initial UDG activation of 50 °C for 2 min and a previous denaturation of 95 °C for 2 min. Those steps were followed for 40 cycles of 5 s of denaturation at 95 °C and 30 s of extension and annealing at 60 °C. The qPCRs were run in duplicate for each gene per sample. The specificity of the q-PCR was confirmed by the detection of a single distinct peak on examination of the dissociation curve profile of the reaction product and the analysis of the amplicon size by agarose gel electrophoresis. The relative gene expression was calculated using the ΔΔCt method. Target gene expression was normalized with the housekeeping reference gene glyceraldehyde-3-phosphate dehydrogenase (*GAPDH*).

### 2.8. Immunofluorescence and Confocal Microscopy

To localize the CXCL12 protein within the in vitro matured COCs, a small cohort of COCs was randomly selected and fixed in 4% paraformaldehyde, washed, and then stored in PBS with 3% BSA at 4 °C until analysis. The COCs were placed in 6-well plates and incubated in a permeabilization buffer (1.5% Triton C-100, 0.15% Tween 20 in PBS) for 1 h at room temperature (RT), in a blocking buffer (1% BSA, 10% Normal Donkey Serum, 0.05% Tween 20 in PBS) for 1 h at RT, and then incubated overnight at 4 °C with the primary antibody (Rabbit anti-CXCL12, LS-B943-100, LSBio, Seattle, WA, USA, 5 μg/mL). The next day, the COCs were rinsed and incubated with the secondary antibody at a 1:200 dilution ratio (goat anti-rabbit IgG H&L, Alexa Fluor 488, ab150077, abcam, Cambridge, UK) for 1 h at RT. After extensive washing (3 × 15 min), the COCs were mounted on slides with 20 μL of Vectashield Mounting Medium (Vector Laboratories, Burlingame, CA, USA) and Hoechst 33342 1:1000 (Thermofisher Scientific, Fremont, CA, USA) as a nuclear stain. Immunofluorescence was validated with a control staining by adding CXCL12 blocking peptide (LS-E10739, LSBio, Seattle, WA, USA).

For CXCR4 immunodetection in spermatozoa, the sperm pellet was fixed in 4% paraformaldehyde for 20 min, and after extensive washing with PBS, 20 μl drops of the sperm suspension were air-dried on poly-l-lysine (0.01 mg/mL)-coated slides. Slides were then washed in PBS-Tween20 (2 × 10 min) and incubated in a blocking buffer (5% BSA in PBS) for 1 h at RT. After washing (2 × 10 min), slides were incubated overnight at 4 °C in a humid environment with the primary antibody (Rabbit anti-CXCLR4, LS-B13299, LSBio, Seattle, WA, USA) at a 1:100 dilution rate. Slides were washed in PBS-Tween20 (3 × 10 min), and then primary antibody binding was visualized by incubating the slides with an Alexa Fluor 488 secondary antibody (goat anti-rabbit IgG H&L, Alexa Fluor 488, ab150077, abcam, Cambridge, UK at a 1:200 dilution) for 3 h at RT. After washing three times in PBS-Tween20 (3 × 10 min), the slides were incubated with 100 μL of Vectashield Mounting Medium (Vector Laboratories, Burlingame, CA, USA) and Hoechst 33342 1:1000 (Thermofisher Scientific, Fremont, CA, USA) as a nuclear stain.

Both the COCs and spermatozoa were evaluated by confocal microscopy (Zeiss LSM800 inverted confocal laser scanning microscope) at ×200 and at ×600 magnification connected to software NIS elements.

### 2.9. Statistical Analyses

Statistical analyses were performed using SPSS statistical software (version 24.0; SPSS Inc., Chicago, IL, USA). The normality of the variables was analyzed by the Kolmogorov–Smirnov. The statistical significance was determined using Student’s *t*-test or ANOVA depending on the number of groups compared. The relative expression of mRNA or protein is expressed as the mean ± the standard error of the mean (SEM). Differences were considered significant at *p* < 0.05. Gene expression values were represented as 2^−ΔΔct^.

## 3. Results

### 3.1. The CXCL12 Gene Is Overexpressed in Mature Cumulus Cells and the CXCR4 Gene Is Overexpressed in Capacitated Spermatozoa

**Figure 1:**
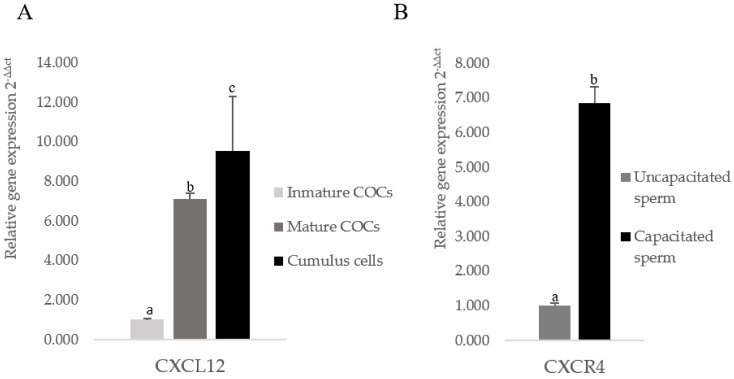
Relative RNA expression of *CXCL12* analyzed in the immature and mature cumulus–oocyte complexes (COCs) and cumulus cell (**A**). Relative RNA expression of *CXCR4* analyzed in incapacitated and in vitro capacitated spermatozoa (**B**). Data are expressed by mean ± SEM. Columns with different superscripts (a and b) and asterisks indicate significant differences (*p* < 0.05) between groups.

We analyzed the relative *CXCL12* mRNA expression in the immature and mature COCs and the cumulus cells from the mature COCs by quantitative RT-PCR. Gene relative expression increased after in vitro maturation, and there were no significant differences between *CXCL12* mRNA expression in the mature COCs and cumulus cells at the same time point (Figure 1A). Boar spermatozoa were subjected to *CXCR4* gene expression analyses by qPCR, and *CXCR4* relative expression was significantly increased after in vitro sperm capacitation compared to uncapacitated permatozoa (Figure 1B).

### 3.2. CXCL12 Is Localized in the Cytoplasm of Cumulus Cells Surrounding Mature Oocytes

**Figure 2:**
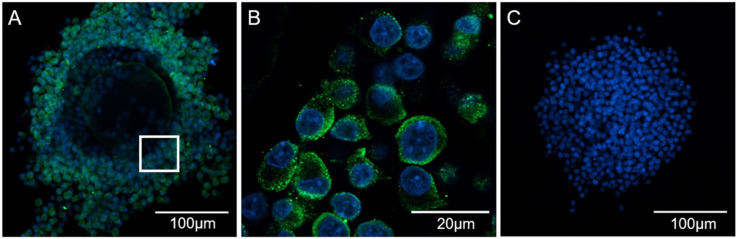
Representative confocal microscopy images of the expression and localization of CXCL12 in porcine mature COCs. From left to right, CXCL12 at different magnifications (**A**,**B**), control staining with blocking peptide showing COCs nuclei (**C**). Blue: nuclei (Hoechst 33342); Green: CXCL12.

We performed immunocytochemical analysis using an anti-CXCL12 antibody and found expression of CXCL12 in cumulus cells. Its signal intensities were localized in the membrane of the cumulus cells surrounding the oocyte (Figure 2A,B). The negative control consisted of samples incubated with the primary antibody and the blocking peptide and exhibited no positive staining (Figure 2C).

### 3.3. CXR4 Is Localized in the Sperm Midpiece and Principal Piece in Uncapacitated Spermatozoa and Also in the Sperm Head of Capacitated Spermatozoa

**Figure 3:**
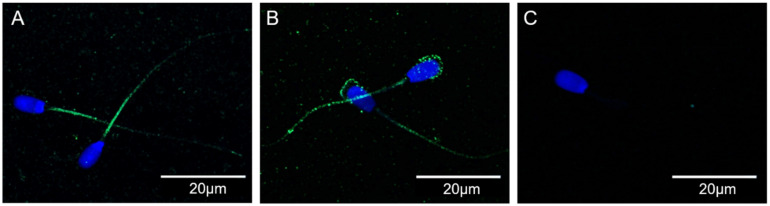
Representative confocal microscopy images of the expression and localization of CXCR4 in spermatozoa. From left to right, CXCR4 in uncapacitated (**A**) and in vitro-capacitated (**B**) spermatozoa, as well as the blocking peptide control, solely showing sperm nuclei (**C**), Blue: nuclei (Hoechst 33342); Green: CXCR4.

The immunolocalization of the receptor CXCR4 was analyzed in uncapacitated and in vitro-capacitated spermatozoa. In the uncapacitated spermatozoa, we detected the presence of CXCR4 in the midpiece and in some small spots on the sperm principal piece (Figure 3A,B), which was prevented by incubation with the primary antibody and the blocking peptide (Figure 3C). Interestingly, CXCR4 was also present in the sperm head of capacitated spermatozoa (Figure 3B). However, there were no remarkable differences in the fluorescence between non-capacitated and capacitated spermatozoa.

## 4. Discussion

The complex mechanism by which only a few spermatozoa reach the oocyte prior to fertilization remains uncertain, and many alternative theories have been presented: from the simple probabilities of chance blocking excessive sperm numbers by female barriers during sperm transport [30] to specific guiding systems, the latter being related to chemoattractants and the presence of diverse contact complexes [31,32]. Sperm chemotaxis has attracted attention as it is considered to be one of the main guidance conditions for spermatozoa to successfully find the oocyte [3,33]. There is a recognized chemokine presence in the female genital tract [25], and a continuous chemokine guidance along the female reproductive tract is considered to be an important prerequisite for spermatozoa to correctly localize the COC [34]. Both the follicular fluid and the cumulus cells are known to secrete a variety of chemokines that promote sperm hyperactivity and increase sperm motility following receptor binding [35,36]. One of the reported cytokines, CXCL12, is able, moreover, to build a complex with CXCR4 (the CXCL12–CXCR4 complex), which has been described as one of the main mechanisms to promote sperm chemotaxis towards the COC [22], although its location in the participating cell types might also represent a specific anchoring mechanism during fertilization, perhaps even selecting/favoring only the capacitated spermatozoa to engage in ZP-binding and further fertilization.

The present study identified that the CXCL12 gene expression was significantly up-regulated in the COCs with a mature oocyte compared to the immature COCs in the porcine species, which is in accordance with previous reports of this species [37,38], as well as in ovine species [23], which points out that CXCL12 signaling is actually contributing to oocyte maturation through the regulation of the expansion of the cumulus cell cloud. Moreover, CXCL12 may play a pivotal role in follicle development in several species, as previously suggested [39]. Recent studies have reported the key roles of CXCL12 in many reproductive processes [40,41] and the assessment of the potential capacity of this chemokine as a sperm attractant [24]. However, to the best of our knowledge, this is the first report describing the localization of this protein within the COC in pigs. Our results show that CXCL12 is produced in the cumulus cells cytoplasm, from where it may act as a sperm chemoattractant prior to fertilization. Previous studies reported that CXCL12 is locally produced by cumulus cells from human [21], cattle [22], and sheep oocytes [23]. Furthermore, besides its role in oocyte maturation, a recent study in cattle [22] suggested that a single COC has the ability to attract spermatozoa towards the oocyte, pointing to CXCL12 as the main sperm chemoattractant candidate, which would interact with its receptor (CXCR4) if localized in the spermatozoa. Interestingly, the sperm-attractant role of CXCL12 may not be necessary to achieve adequate penetration and fertilization rates during in vitro fertilization, where the cumulus cells are removed from the oocytes after in vitro maturation. However, the proportion of spermatozoa (1000–2000 spermatozoa/oocyte) used in the majority of IVF protocols [42] is much higher than that found in the oviduct during physiological fertilization [43]. Thus, it seems reasonable to think that several mechanisms triggered towards gamete-encountering are only activated during in vivo fertilization.

The CXCL12-CXCR4 signaling is able to induce sperm hyperactivation by increasing sperm intracellular calcium, probably by regulating the function of Catsper channels [21]. These phenomena seem to reflect spermatozoa chemotaxis, and that CXCL12-CXCR4 signaling could represent an important event during oocyte fertilization.

To date, CXCL12 is the only known natural ligand for CXCR4 [44,45]. In the present study, CXCR4 was found to be significantly up-regulated in capacitated spermatozoa compared to uncapacitated spermatozoa. This finding suggests that the activation of the CXCL12-CXCR4 signaling occurs closely prior to gamete encounter, when both the oocyte and the spermatozoa are prepared to achieve fertilization. In addition, we identified CXCR4 protein in the midpiece and principal piece of the porcine sperm tail in uncapacitated spermatozoa, but also in the sperm head after in vitro capacitation. The latter might well mean that the complex might be relevant in favoring the entry of spermatozoa in the COC cloud and allowing capacitated spermatozoa to reach the ZP.

It is widely known that proteins are subjected to a redistribution within the sperm membrane during the capacitation process in anticipation of acquisition of fertilizing ability [46,47]. Our findings suggest that CXCL12-CXCR4 signaling may also induce sperm hyperactivation, adding a mechanism for an earlier stage of guidance followed in capacitated spermatozoa by a further sperm head guidance towards the ZP. It appears that the localization of this protein in the spermatozoa is species-related. In humans, CXCR4 has been identified in the sperm acrosome [24,48], while it has been described in the sperm midpiece and flagella in cattle [22]. This is the first report to describe the localization of the protein CXCR4 in the porcine spermatozoa, with differential localization in relation to the status of hyperactivation/capacitation.

## 5. Conclusions

In conclusion, the present study shows that *CXCL12* and *CXCR4* gene expression is significantly increased in mature cumulus cells compared to immature COCs and in capacitated spermatozoa compared to uncapacitated spermatozoa, respectively. Moreover, we described for the first time that the CXCL12 protein is located in the cytoplasm of the cumulus cells from mature COCs, and that the CXCR4 protein is expressed in the midpiece and principal piece of uncapacitated spermatozoa and also in the sperm head after in vitro capacitation in the porcine specie. These findings increase our current knowledge on porcine physiology of fertilization and reproduction, leading to possible improvements in the performance of reproductive technologies.

## Figures and Tables

**Table 1:** Primer sequences for the analysis of mRNA gene expression.

GENE	Forward (5′→3′)	Reverse (5′→3′)	Size	Efficiency (%)	Reference
*CXCL12*	TAAACAAACCCAGTCCCACTCTC	AGGAAATAAACATCCCGCCGT	115	94.1	NC_027311.1
*CXCR4*	TGGACGGGTTCCGTATATTCAC	GAAATGGGCATTTTCCTCCCG	110	93.4	NC_010457.5
*GAPDH*	ATCACTGCCACCCAGAAGAC	AGATCCACAACCGACACGTT	194	96	NM_001206359
